# Equine Metabolic Syndrome: A Complex Disease Influenced by Multifactorial Genetic Factors

**DOI:** 10.3390/genes14081544

**Published:** 2023-07-27

**Authors:** Monika Stefaniuk-Szmukier, Katarzyna Piórkowska, Katarzyna Ropka-Molik

**Affiliations:** Department of Animal Molecular Biology, National Research Institute of Animal Production, Krakowska 1, 32-083 Balice, Poland

**Keywords:** equine metabolic syndrome, horses, insulin resistance, obesity, management

## Abstract

Equine metabolic syndrome (EMS) has become an important issue in modern veterinary medicine and is linked to the common, extremely painful, most-of-the-time performance-terminating hoof laminitis. The growing knowledge in the field of genetic background, inducing environmental factors, diagnosis, treatment and maintenance of affected equines led us to summarise the available information to be used not only for scientific purposes but for fieldwork. In horses, the clinical presentation of EMS includes: obesity or local fat deposition, bilateral lameness or hoof rings attributed to ongoing or previous (pasted) laminitis with the key feature of the occurrence of insulin dysregulation, disturbing the homeostasis within insulin, glucose and lipid metabolism. The management of EMS is based on dietary and fitness discipline; however, intensive research is ongoing in the field of regenerative medicine to develop modern and promising therapies.

## 1. Introduction

The concept of Equine metabolic syndrome (EMS) was introduced in 2002 by Johnson [1] and gained wide acceptance as defining a unique syndrome in horses. It was proposed that obesity, insulin dysregulation, and laminitis are elements of this clinical disorder syndrome. However, recent findings have suggested a connection between obesity and EMS (GEES 2022). The term EMS was adopted because of its similarity to human metabolic syndrome, which is a set of risk factors mainly for the assessment of coronary heart disease and type 2 diabetes [2]. The term EMS was agreed upon because it has gained wide acceptance and is suitable to define a unique clinical syndrome in horses. In the meantime, other terms have been used to describe EMS, such us prelaminitic metabolic syndrome, syndrome X, insulin resistance syndrome, obesity-associated insulin resistance (IR) and prediabetes mellitus. 

Insulin is a key systematic anabolic peptide hormone that maintains the homeostasis of carbohydrates, proteins and lipids. It is secreted by β cells of the islets of Langerhans in the endocrine part of the pancreas. Insulin binds to specific receptors (IRS), activating a downstream signalling cascade with the role of phosphorylation as a serine/threonine kinase PKB/Akt. PKB/Akt inhibits lipolysis via PDE3, which, in turn, inhibits cAMP and reduces the phosphorylation of HSL (hormone-sensitive lipase) and, therefore, its lipolytic activity. Moreover, insulin stimulates lipogenesis and increases fatty acid synthase (FAS). The glucose uptake by insulin-dependent tissues begins when the translocation of GLUT 4 to the plasma membrane occurs from the intracellular pool. This process is insulin-dependent, associated with the activation of kinase PI3K and it increases the glucose availability for glycolytic transformation and glycogen synthesis by activating glycogen synthase (GS) [3,4]. 

## 2. Insulin Dysregulation: A Central Hub

After years of research, the present understanding of the pathophysiology of the risk factors involved in the occurrence of the syndrome indicates that insulin dysregulation plays a key role in EMS. Insulin dysregulation can be manifested in basal hyperinsulinemia, which is abnormal hyperinsulinemia in response to carbohydrate administration (oral or intravenous) or insulin resistance [5]. It is worth mentioning here that factors affecting the reference values for the basal insulin levels could be pregnancy, high-energy forage, stress and illness, as well as low-carbohydrate diets, poor forage with little grain or anti-inflammatory medications and certain antibiotics. Additionally, recent suggestions have also indicated that IR has a gastrointestinal aetiology. 

Insulin dysregulation can be manifested by hyperinsulinemia. In the recent literature, there have been some doubts expressed about insulin concentration measurements, mostly because of the sampling collection and feeding. However, the consensus statement of the American College of Veterinary Internal Medicine recommends sampling prior to feeding. Fasting hyperinsulinemia is associated with the sustained stimulation of pancreatic β cells, elevated fatty acid concentrations between meals in obese IR individuals, β-cell hyperplasia and insulinoma. However, the measurement of postprandial insulinemia (PI) in equids is used despite disagreement about the length of feeding and digestion and the variation in feed composition. PI might be due to increased insulin secretion or delayed insulin clearance, as a result of IR, when decreased insulin causes tissue sensitivity. Nevertheless, cases of equines with postprandial hyperinsulinemia with normal glucose and insulin responses are known. Other than IR, possible causes of PI following the ingestion of sugars are hormonal disorders on the entero-insular axis (EIA) [6].

Two hormones play the main physiological role in enhancing insulin levels after their nutrient-induced secretion from the gut: the incretin hormone glucose-dependent insulinotropic polypeptide (GIP) and glucagon-like peptide-1 (GLP-1). GIP is a peptide hormone consisting of 42 amino acids and acts in the gut–endocrine–pancreas axis (enteroinsular axis—EIA) as an anabolic hormone, increasing insulin levels and stimulating glucagon secretion. In turn, the GLP-1 gene encodes 30 amino acid peptides produced in intestinal epithelial endocrine L cells. The main functions of GLP-1 are to stimulate insulin secretion and inhibit glucagon secretion, thereby limiting postprandial glucose levels [7,8]. These two incretin hormones have overlapping biological roles; thus, within the EIA, they maintain physiological glucose tolerance, pointing, presumably, to the functional involvement of the entero-insular axis (EIA) in insulin dysregulation, owing to predisposition to laminitis. A study performed on a group of horses with different statuses of insulin dysregulation found that IR is not essential to the hyperinsulinemia that predisposes horses to laminitis, and gastrointestinal factors play a role in ID, as horses with excess insulin sensitivity absorb more and metabolize less glucose from their diet than physiologically normal horses. Furthermore, almost 23% of the variation in insulin concentrations can be explained by the aGLP-1 concentrations after the oral administration of non-structural carbohydrates (NSCs) [9]. Additionally, it has been shown that ponies with ID had higher postprandial insulin responses compared with healthy ones while grazing the same pasture [10]. Insulin resistance (IR), in general, occurs when tissues show reduced sensitivity to insulin despite a normal or even decreased concentration in the blood, and the consequences can be impaired glucose uptake by the tissues, increased efficiency of gluconeogenesis and increased lipolysis. With IR, the peripheral tissues are unable to increase glucose uptake from the blood. At the initial stage, the β cells of the pancreas secrete elevated levels of insulin, consequently leading to hyperinsulinemia, but glucose is still transported to the cells [11]. Moreover, insulin resistance per se is not necessarily a precursor of hyperinsulinemia and, finally, laminitis.

## 3. The Primary EMS Consequence: Laminitis

Laminitis is a multifactorial condition where disruption of the distal phalangeal suspensory apparatus at the dermal–epidermal junction occurs. It is associated with microanatomical lesions of the epidermal and dermal lamellae that result in structural failure of the suspensory apparatus. Four phases of the condition have been distinguished: developmental acute, subacute, chronic and endocrine. Metabolic diseases are considered to be some of the most common causes of laminitis [12].

Four different mechanisms have been proposed that ultimately lead to laminitis: inflammation and degradation of the extracellular matrix of the hoof lamellae, endothelial or blood vessel dysfunction, hormonal or metabolic disorders and mechanical overload [13]. Subsequent laminitis phases can be divided into risk of onset, development (first symptoms) and the clinical stage. The first stage occurs when animals are exposed to risk factors or triggers such as EMS [14]. The first symptoms of laminitis are usually not detected, and the cascade of pathological changes that cause damage to the lamellae lasts for hours or even days. The clinical stage can be divided into acute, subacute and chronic. The acute stage includes a characteristic shifted/bearing posture with relieving heels, separation of the hoof wall and hoof bone rotation [15]. The subacute form is associated with mild episodes or undetected episodes that are noticeable only after some time in the form of rings on the wall of the hoof. Laminitis causes changes in the blood flow to the hoof, increased expression of cytokines that activate inflammation and altered glucose metabolism within the lamellae [16]. 

To determine if laminitis will result after prolonged hyperinsulinemia, healthy horses were treated with intravenous insulin. As a result, all experimental horses developed laminitis with early clinical symptoms (after 31.5 h), including a significantly increased heart rate, anxiety and episodic shifting of the feet. After the next few hours, the horses had more consistent shifting of weight, had a stiff gait at walking and were reluctant to trot [17]. 

Endothelial cells containing insulin receptors activating nitric oxide synthase cause increased nitric oxide (NO) production, which, in turn, has a vasodilation effect—it dilates the blood vessels and, thus, increases the perfusion in the hoofs, which may be one reason for laminitis [18]. Additionally, a large amount of the glucose transporter-1 (GLUT-1) glycoprotein has been confirmed within the lamellae. GLUT-1 is responsible for active glucose transport into the cell, which indicates that this structure can receive glucose independently of insulin [19]. It was suggested that despite normal glucose concentrations, local fluctuations or increased uptake by GLUT-1 transport proteins is possible as a result of increased perfusion, which leads to the degradation of the lamellae [17]. 

Many EMS horses experience mild subclinical attacks before they are properly diagnosed. Studies of the hormonal and metabolic status of animals affected by chronic laminitis determined that hyperinsulinemia is their common feature [20,21,22]. In addition, the degree of hyperinsulinemia was correlated with the severity of laminitis [23,24] and was predicted for previous episodes in animals with elevated insulin levels [25]. The histological study of insulin-induced laminitis suggests a mitogenic effect of insulin, which confirms the in vitro observation that epithelial cells of the lamina proliferate during incubation with insulin [26]. However, insulin receptors do not occur on the surface of the lamellar epithelium; therefore, insulin may exert its action through insulin-like growth factor 1 (IGF-1) receptors that are present on the surface of the epithelial cells of the lamellae. IGF-1 has a high degree of structural homology with insulin, and there are some similarities between these hormones and their receptors, although with much lower affinity and lower binding power, insulin may exert its effect on endothelial cells in the lamellae by binding to IGF-1 receptors [27,28]. Because of the lamellae’s high demand for glucose, the deprivation of glucose access due to hyperinsulinemia has been mentioned as a possible mechanism for inducing laminitis [29]. However, it has been shown that glucose uptake is carried out via glucose-independent transporters, and changes in glucose level may cause laminae insufficiency [13,30] through the accumulation of final glycation products (AGEs), which, in turn, could damage tissues during sporadic or persistent hyperglycaemia, as observed in humans [31]. An ex vivo model of laminitis supported the hypothesis that methylglyoxal (MG), which causes the formation of AGEs derived from glucose, led to the hoof lamellae losing the mechanical properties of the response to the application of force. The *MMPs* and *TIMP-2* gene expression study indicated that increased levels of MMPs are correlated with increased extracellular matrix remodelling and, thus, structural weakness associated with hoof lamellae separation [32]. The matrix metalloproteinases play a main role in the cleavage of the extracellular matrix, leading to selective degradation of membrane proteins and collagens. The impact of abnormal glucose levels may not be a directly reflected in the lamellae; however, insulin resistance may lead to changes in blood circulation inside the hoof. The dysfunction of numerous arteriovenous fusions has been described as a mechanism contributing to the development of laminitis [17]. In vitro studies revealed that incubation of the finger vessels in high concentrations of insulin results in the loss of their ability to expand and vessel hypertension in insulin-resistant horses [22,33]. In conclusion, laminitis in horses is a consequence of vascular dysfunction resulting from a metabolic syndrome where a high concentration of insulin through dysfunction of the vascular endothelium affects the blood supply to the hoof. 

## 4. Obesity Implications as a Secondary Consequence of EMS

In the past, the probability of EMS was mostly predicted via phenotypical observations supported by the measurement of the cresty neck score together with the characteristic of adipose tissue deposition, this circumference along with the ratio of the circumference of the neck to the height at the withers, body weight based on the scale or zoometric tape, as well as body condition scoring (BCS) [34]. However, the ECEIM consensus statement on the occurrence of EMS diagnosed based on obesity is very misleading [5]. The implication of obesity influencing the emergence of EMS was based on the hypothesis that the supply of too much energy exceeded the demand through an inappropriate diet, rich in non-structural carbohydrates. Feeding with high-feed fodder and high-quality hay, where energy is concentrated in a much smaller dietary dose, has significantly changed the natural eating habits of horses, which are evolutionarily adapted to the collection of inferior-quality feed [35]. Consequently, increased fat accumulation enlarges the size of adipocytes which, in turn, experience cellular stress because of endothelial dysfunction and poor oxygen diffusion. Their inability to absorb excess lipids may also contribute to the ectopic deposition of lipids and lipotoxicity in other body tissues. The adipose tissue synthesizes and secretes biologically active adipokines that can regulate, e.g., energy metabolism, and cardiovascular and immune functions [36]. 

One of the best-known equine adipokines is leptin, whose main function is regulation of the energy metabolism. Leptin acts via regulation of the satiety centre in the hypothalamus to inhibit food intake and increase energy expenditure through thermogenesis. In horses, serum leptin concentrations were positively correlated with obesity [37,38,39]. It was determined that hyperleptinemia (>7.3 ng/mL), as well as hyperinsulinemia (>32 mIU/L), may help predict laminitis in ponies [40]. Furthermore, the level of leptin expression is increased in nuchal ligament adipose tissue, suggesting that local fat accumulation at this site can significantly contribute to elevating the overall level of leptin in horses [41]. The pathologically deposited adipose tissue becomes metabolically active and begins to produce hormonal and proinflammatory factors that disturb the proper working of insulin on elevated glucose after feeding [42]. The relationship between obesity and insulin-level disorders in equids has been described in numerous cross-sectional studies [20,40,43,44]. A controlled weight gain experiment on Arabian horses and geldings of crossbred Arabians was performed. The study design provided 200% of the daily requirement for digestible energy for 16 weeks. The experimental animals increased body weight by 20%, and their insulin sensitivity was reduced by 70%. These results led to the consideration that obesity might be recognized as a factor modifying individual predisposition to insulin dysregulation in horses. Furthermore, it has been shown that feeding horses with a diet rich in sugar and starch decreases insulin sensitivity and increases the evolving metabolic disorders associated with the maintenance of body condition and feeding management [43]. The effect of adaptation to high-glycaemic meals on glucose–insulin regulation in thoroughbred weanlings indicated that highly glycaemic feed alters insulin sensitivity, which may result in further insulin resistance [44]. 

The approach to the relationship between EMS and obesity is constantly evolving because of the significant dysfunction of adipocyte tissue (both perirenal and retroperitoneal) in EMS horses (adipocyte hyperplasia; overexpression of leptin and cytokines) [45].

It has been shown that EMS has a negative impact on the adipose-derived stem cells (ASCs) of affected horses. The surface marker expression of *CD44+* is significantly higher and stimulates the inflammatory reaction in adipose tissue. The adipose-derived stem cells derived from EMS affected horses (ASCems) displayed a higher number of mitochondrial abnormalities, high nitric oxide (NO), higher reactive oxygen species (ROS) production and accumulation of oxygen species, together with reduced dismutase (SOD) activity, indicating that under oxidative stress toxic compounds accumulated. Furthermore, in ASCems deregulated the expression of molecular markers related to apoptosis, autophagy, regenerative ability, elimination of dysfunctional mitochondria and level of metabolism and ATP production in cells, such as *p21*, *Bcl-2/BAX*, *BMP-2*, *PGC1α* and *PARKI*; *PDK4* indicated dysfunction of the mitochondria [46]. Because of oxidative stress, apoptosis and mitochondrial function deterioration, the ASCems possess a senescent phenotype with enlarged cell bodies and nuclei, increased apoptosis and reduced heterochromatin architecture. However, the ASC EMS-affected cells develop autophagy shifts during an early stage of differentiation to maintain cellular homeostasis, allowing for the removal of ROS-induced changes [46]. Further investigation of other tissues of EMS horses showed that adipose and liver tissues are affected by stress induced via p53 signalling endoplasmic reticulum stress and autophagy, which, in turn, protects the cells against glucolipotoxicity. Interestingly, in EMS muscles, other mechanisms must occur to protect cells because of insulin-dysfunction-related apoptosis [47].

As obesity is a common phenotypic indicator in horses with EMS, some studies have suggested that in obese and insulin-resistant horses, the liver may play an important role. The liver impairment in EMS horses at the molecular level is manifested by increased apoptosis and increased endoplasmic reticulum, oxidative stress, excessive accumulation of lipids and increased inflammation. Several studies in the field of regenerative veterinary medicine reported the possibility of using the transplantation of modified autologous adipose stem cells to reduce the impairment of the liver [48].

## 5. Genetic Aspect of Equine Metabolic Background

The genetic component of EMS was suggested in several studies and led to further investigation of the genetic background of the syndrome. The evaluation of markers seems to be a promising tool for the diagnosis of predisposed horses and decreases the cost of diagnosis as well as early detection of affected horses. Evaluating genetic and metabolic predispositions to EMS involved pasture-associated laminitis in ponies; a herd kept on pasture was evaluated for episodes of laminitis, concentrations of glucose, triglycerides, non-esterified fatty acids, insulin concentrations, body condition scores and pedigree. Additionally, pastures were sampled for analysis of simple sugars and starch. Results of the study pinpointed that ponies predisposed to pasture-associated laminitis present different metabolic profiles in terms of insulin resistance, compensatory hyperinsulinemia and glucose and fat metabolism. The pedigree evaluation and association with the prevalence of laminitis suggested a dominant pattern of inheritance of a major gene or genes with reduced penetration owing to sex, age of onset and possible epigenetic modifications [21]. Furthermore, it was also determined that ponies are twice as likely to develop pasture-associated laminitis than other breeds of horses [49]. McCue et al. proposed a genetic scenario/schema underlying EMS. Phenotypic components of EMS consist of mechanisms involved in the regulation of insulin, glucose and lipid metabolism that are part of complex metabolic pathways with multiple and numerous alleles, which, in turn, are influenced by epigenetic modification under environmental pressure. Following this track, the genetic background responsible for the risk of EMS is a unique combination of several loci. This assumption can also be used to understand the breeds’ differences in susceptibility to EMS [50]. Furthermore, the complex model of EMS susceptibility might be used to explain breed differences in morbidity in EMS. According to this hypothesis, all suspected breeds share two major alleles responsible for hyperinsulinemia and hypertriglyceridemia, and each breed also has a unique combination of modifying alleles with different effects (alleles responsible for insulin release, tissue utilization and lipolysis, enhanced insulin-sensitivity-elevated NEFAs). 

The high-throughput methods allowed for investigation of the polygenetic character of EMS in a more comprehensive way. A recent paper by Lewis et al. analysed the potential genetic background of the occurrence of EMS in Arabian horses using the Equine SNP50 Beadchip. The Genome-wide association studies (GWASs) approach revealed three promising genetic markers (BIEC2-263370, BIEC2-263373 and BIEC2-263524) that potentially influence the risk of laminitis occurrence, associated with elevated insulin levels, together with insulin resistance and the horse condition evaluation index. SNPs are located within the *FAM174A* gene region. Nevertheless, *FAM174A* (also known as *NS5ATP6*; *TMEM157*) may play a lipid regulatory role. Research has shown a strong and significant correlation between genotype and biochemical and lipid blood parameters: horses with BIEC2-263524 and *FAM174A* risk alleles were characterized by increased triglycerides content, while horses with the BIEC2-263524 mutant variant had elevated cholesterol levels [51]. 

Moreover, studies performed in human medicine proved that the knockdown of *FAM174A* in HeLa K cells results in the downregulation of low-density lipoproteins (LDLs) and increases cellular-free cholesterol [52]. Further studies indicated that FAM174A does not affect total intracellular cholesterol (TC) levels in in vitro studies, but the deregulation of *FAM174A* results in changes in triglycerides content (TG) [53]. However, the most important finding seems to be an interaction between *FAM174A* and *FGF21*. There is growing evidence that fibroblast growth factor 21 plays a crucial role in the regulation of energy metabolism, enhances insulin sensitivity, acts as an activator of glucose uptake on adipocytes, protects against diet-induced obesity and is a crucial mediator of the adaptive starvation response [54]. It was proved via an *FGF21* promoter luciferase assay that *FAM174A* transcriptionally regulates *FGF21*. Furthermore, investigation of other regulatory possibilities revealed overexpression of *FAM174A*-upregulated miR-577 at the post-transcriptional level [53]. *FAM174A* has the potential to influence EMS because of its interaction with *FGF21*. Furthermore, a study involving a Polish population of Arabian horses revealed a high level (51%) of one of the potential makers within *FAM174A* (BIEC2-263524) correlating with higher insulin levels and laminitis {Formatting Citation}. The breed-specific relationship between genetic factors and the occurrence of EMS was confirmed by a study on Australian ponies with a known metabolic status. The authors questioned the strong association of the *FAM174A* gene with EMS in this breed because of the low frequency of the risk allele within all affected individuals. The horses with EMS syndrome and the identified *FAM174A* polymorphism accounted for only 15% of the total affected population. Moreover, most of the affected ponies showed obesity, but there was no association between obesity and *FAM174A* genotypes [55]. The result differed from the one presented in Arabian horses, where obesity was proposed as one of the main phenotypic features related to the *FAM174A* risk allele [56]. A recent report indicated that in horses, overweight and obesity may not necessarily be associated with EMS occurrence [5].

The risk of EMS occurrence, also in terms of genetic factors, was evaluated in a large cohort of Morgan horses and pony breeds. Application of GWAS analyses (Illumina SNP50 chip) on 286 Morgan horses allowed for narrowing down the search area to 17 genome-wide significant candidate loci for nine metabolic traits and two morphometric measurements. The top-ranked QTL for the investigated breeds was located within 1MB of *ZFAT* and *HMGA2* genes, while genes overlapping with the human metabolic syndrome were *VEGFA, NRXN3, GRIK2, TRIB2, AHR* and *ISL*, potentially related to EMS risk. Notably, the *AHR* gene coding for the aryl hydro-carbon receptor was also associated with the regulation of energy metabolism and metabolic syndrome in humans. The genome-wide significant loci for fasting glucose were near the *CRYBA4* and *DLGAP4* genes, and those for fasting insulin levels were near *GRIK2* and *ATG14*. The GWAS results for the glucose levels 75 min post-oral sugar test indicated the *SPAG16* and *HDAC1* genes, while the insulin levels 75 min post-oral sugar test indicated the *ISL1* and *RNF217* genes [57,58].

A study conducted on Morgan (*n* = 286) and Welsh ponies (*n* = 264) estimating the SNP-based heritability (h2 SNP) of nine traits measured according to EMS diagnosis (glucose, glucose-OST, insulin, insulin-OST, NEFA, serum concentrations of triglycerides, adiponectin, leptin, ACTH) showed that, in Welsh ponies, seven biochemical parameters reached significant values at the top of ACTH concentration levels. In Morgan horses, six of the analysed traits had significant estimates that excluded glucose concentrations, TG and ACTH. This finding was strong evidence of a genetic contribution to EMS factors, also indicating differences among breeds. Further studies conducted on Welsh ponies demonstrated that baseline insulin levels are correlated with height. This relationship has been well described in humans. People of shorter stature are at risk of developing metabolic diseases, in particular, type 2 diabetes. The correlation analyses between height in ponies and several biochemical parameters indicated a significant inverse correlation between insulin, glucose, adiponectin and ACTH levels and a positive correlation between height and triglycerides and leptin concentrations. In a genome-wide association study, two loci on the equine 6 chromosome roughly contributed to 40% and 20% of height and insulin levels, respectively. Deeper analysis pointed to two genes, *HMGA2* and *IRAK*, for which an *HMGA2*:c.83G>A variant (p.G28E) is a candidate gene with a pleiotropic effect [59]. The *HMGA2* gene (High-Mobility Group AT-Hook 2) encoding protein contains three AT-hook DNA-binding motifs, acts as a transcription regulator and is involved in adipogenesis and diet-induced obesity [59]. In Shetland ponies, the *HMGA2*:c83G>A variant was associated with a reduction in overall size [60]. 

Since the phenotypic manifestation of the characteristics associated with equine metabolic syndrome is associated with significant economic losses, including the deaths of horses, the search for solutions allowing for early diagnosis is crucial. Such tools are at the disposal of modern genetics, so joint efforts to precisely phenotype predisposed animals and identify the genetic variants underlying this complex syndrome seem to be the most important challenge. Thus, further studies on the genetic involvement of EMS occurrence in horses will be a significant step toward understanding the genetic background of risk factors in several breeds.

## Data Availability

No applicable.
